# Quantitative Ultrasound Imaging and Artificial Intelligence in Neonatal Echocardiography: Methodological Advances, Reproducibility Challenges, and Computational Perspectives

**DOI:** 10.3390/jimaging12080356

**Published:** 2026-08-05

**Authors:** Aikaterini I. Nikolaou, Nikitas Chatzigiannis, Maria Alexandra Kefala, Eleni Papaioannou, Maria Baltogianni, Lida-Eleni Giaprou, Vasileios Giapros

**Affiliations:** 1 Neonatal Intensive Care Unit, School of Medicine, University of Ioannina, 45500 Ioannina, Greece; nikaikaterini@gmail.com (A.I.N.); nikihatzi@gmail.com (N.C.); mariakefala2004@gmail.com (M.A.K.); mbalt@doctors.org.uk (M.B.); md07243@uoi.gr (L.-E.G.); 2 Neonatal Intensive Care Unit, Hippokration Hospital, Aristotle University of Thessaloniki, 54642 Thessaloniki, Greece; epapaioannu@gmail.com

**Keywords:** neonatal echocardiography, artificial intelligence, machine learning, deep learning, cardiovascular ultrasound, speckle-tracking echocardiography, spatiotemporal image correlation, quantitative imaging biomarkers, congenital heart disease, neonatal intensive care unit

## Abstract

Neonatal echocardiography remains an essential imaging modality for the assessment of congenital and hemodynamic cardiovascular abnormalities in critically ill neonates. Recent advances in quantitative ultrasound biomarkers, deformation imaging, volumetric reconstruction, and artificial intelligence (AI)-assisted analysis have substantially expanded the diagnostic capabilities of neonatal cardiovascular ultrasound imaging. This narrative review critically examines current developments in quantitative echocardiographic imaging, advanced volumetric methodologies, and AI-assisted cardiovascular ultrasound analysis, with emphasis on neonatal intensive care applications. A literature search was conducted using PubMed, Google Scholar, Scopus and Embase focusing on neonatal echocardiography, spatiotemporal image correlation (STIC), speckle-tracking echocardiography, artificial intelligence, machine learning, and quantitative cardiovascular imaging. Recent studies suggest that advanced methodologies, including speckle-tracking echocardiography, STIC-based reconstruction, automated segmentation algorithms, and deep learning frameworks, may improve image standardization, automated quantification, and congenital heart disease detection. However, important challenges persist, including operator dependency, dataset heterogeneity, limited external validation, cross-platform variability, and incomplete integration into routine neonatal intensive care workflows. Most AI-assisted echocardiographic systems remain investigational and require further prospective multicenter validation before widespread clinical implementation can be achieved.

## 1. Introduction

Congenital heart disease (CHD) remains the most common congenital malformation worldwide and a major cause of neonatal morbidity and mortality, with many affected neonates requiring early cardiovascular intervention [1,2]. Neonatal echocardiography constitutes a primary imaging modality for the diagnosis and functional assessment of congenital and hemodynamic cardiovascular abnormalities because of its non-invasive nature, bedside applicability, and ability to provide real-time hemodynamic evaluation in critically ill neonates [3,4]. Over the past decade, targeted neonatal echocardiography (TNE) has evolved into an increasingly specialized hemodynamic imaging discipline supported by standardized imaging protocols and formalized training recommendations [5,6].

Despite substantial technological advances in ultrasound systems, neonatal and fetal echocardiography remain highly operator-dependent imaging modalities characterized by significant technical and interpretative variability [2,7]. Reproducible cardiovascular ultrasound assessment in neonatal and fetal populations is challenged by small cardiac dimensions, elevated heart rate, restricted acoustic windows, and motion-related image degradation [2,8]. Consequently, modern neonatal echocardiography increasingly incorporates quantitative imaging biomarkers and deformation imaging techniques to improve objective functional and hemodynamic assessment [3].

Although several advanced imaging technologies, including spatiotemporal image correlation (STIC) and Fetal Intelligent Navigation Echocardiography (FINE), were originally developed for fetal cardiovascular imaging, they are discussed in this review because they represent important methodological foundations for subsequent computational advances in neonatal echocardiography. While their direct clinical application remains predominantly prenatal, the underlying principles of volumetric reconstruction, automated plane recognition, and AI-assisted image analysis have substantially influenced the development of neonatal cardiovascular imaging methodologies.

In parallel, the transition from conventional two-dimensional echocardiography toward advanced volumetric imaging methodologies, including spatiotemporal image correlation (STIC) and Fetal Intelligent Navigation Echocardiography (FINE), has expanded the diagnostic capabilities of fetal and neonatal cardiovascular ultrasound imaging [1,9,10]. The increasing complexity of quantitative and volumetric imaging has accelerated the integration of artificial intelligence (AI), machine learning (ML), and deep learning (DL) methodologies into cardiovascular ultrasound analysis workflows [8,11]. AI-assisted echocardiographic systems have demonstrated promising performance in proof-of-concept and early validation studies across several applications, including automated plane recognition, chamber segmentation, myocardial functional quantification, and congenital heart disease detection [8,12].

Nevertheless, important methodological and translational challenges continue to limit widespread implementation of AI-assisted neonatal cardiovascular imaging, including limited dataset size, inadequate external validation, annotation inconsistency, and incomplete integration into clinical workflows [7,13]. The aim of this review is to critically examine current advances in quantitative neonatal echocardiographic imaging, focusing on deformation imaging, volumetric ultrasound methodologies, artificial intelligence-assisted analysis, reproducibility limitations, and future directions in neonatal cardiovascular ultrasound assessment.

## 2. Materials and Methods

This study was conducted as a structured narrative review to critically examine recent advances in quantitative imaging, volumetric ultrasound methodologies, and artificial intelligence (AI)-assisted analysis in neonatal echocardiography. A comprehensive literature search was performed using PubMed, Scopus, Embase, and Google Scholar to identify relevant studies published between 2010 and May 2026. The search strategy combined controlled vocabulary, where applicable, and free-text terms using Boolean operators. Representative search terms included “neonatal echocardiography”, “targeted neonatal echocardiography”, “fetal echocardiography”, “cardiovascular ultrasound”, “artificial intelligence”, “machine learning”, “deep learning”, “speckle-tracking echocardiography”, “spatiotemporal image correlation (STIC)”, “deformation imaging”, “quantitative imaging biomarkers”, and “congenital heart disease”. Reference lists of relevant publications were also manually screened to identify additional studies.

**Figure 1:**
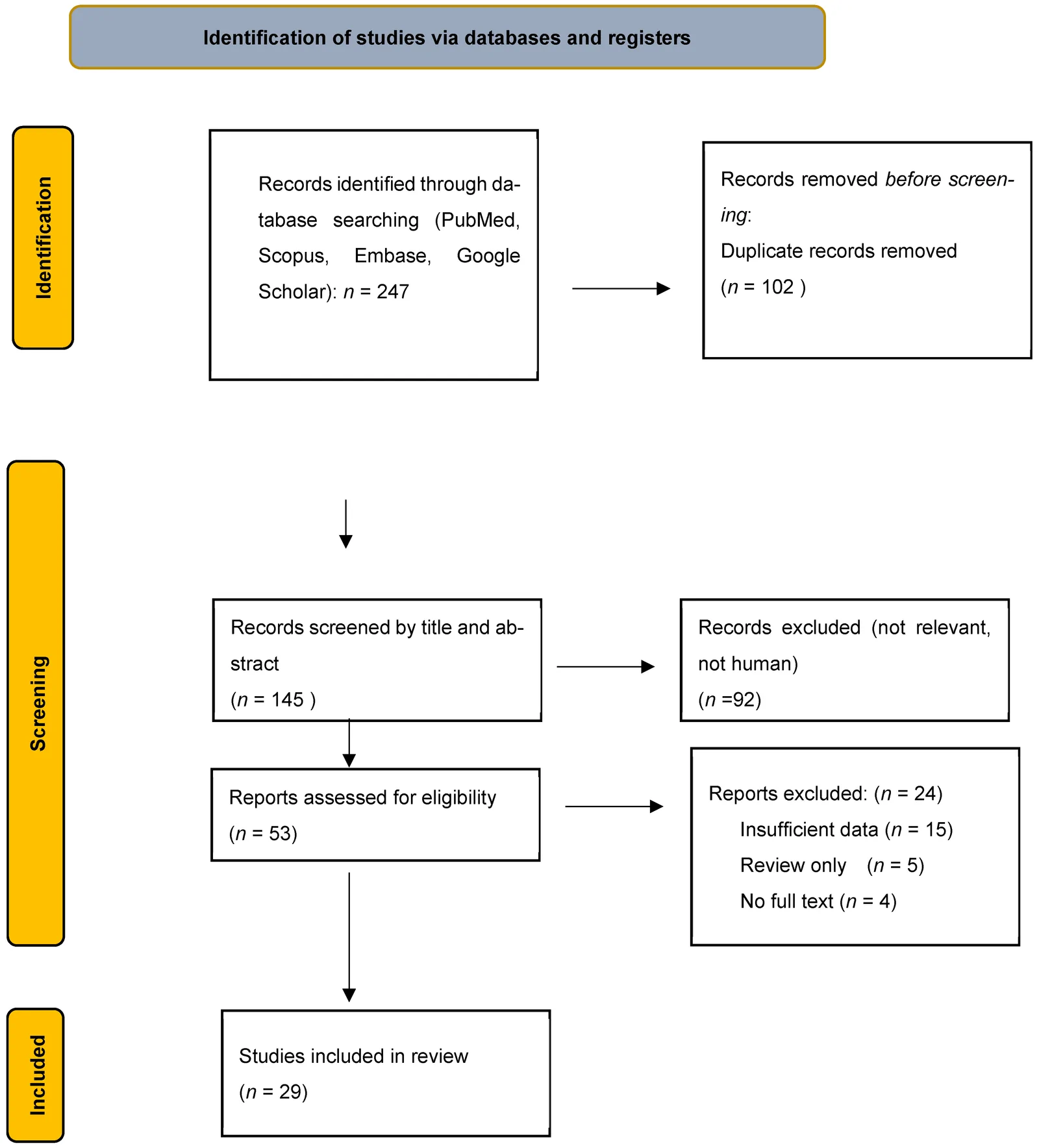
PRISMA flow diagram of search procedure.

Studies published in English were considered eligible if they focused on neonatal or fetal echocardiography, quantitative cardiovascular ultrasound imaging, volumetric reconstruction, or AI-assisted echocardiographic analysis. Editorials, letters, duplicate publications, and case reports without substantial methodological contribution were excluded. Following title, abstract, and full-text screening according to the predefined eligibility criteria, 29 studies were selected from an initial pool of 247 records based on their relevance to the objectives of this review. Study identification, screening, and eligibility assessment were initially conducted by the first author. The final selection of eligible studies was independently reviewed by the senior author, and any uncertainties regarding study eligibility were resolved through discussion and consensus. The study selection process is illustrated in the PRISMA flow diagram (Figure 1), while the principal elements of the search strategy, eligibility criteria, and evidence synthesis are summarized in Table 1.

The selected studies were synthesized narratively and critically, with particular emphasis on study methodology, reproducibility, external validation, and clinical applicability of the reported findings. As this work represents a structured narrative review rather than a systematic review or meta-analysis, no formal risk-of-bias assessment or quantitative meta-analysis was performed. Nevertheless, methodological quality, clinical relevance, and external validation were considered during evidence synthesis and interpretation to ensure a balanced and evidence-based synthesis of the available literature.

## 3. Technical Challenges and Methodological Limitations of Neonatal Echocardiography

Neonatal echocardiography presents important technical challenges because of small cardiac anatomy, elevated heart rates, rapid hemodynamic transitions, and limited acoustic access during intensive care monitoring [3]. High neonatal heart rate substantially reduces the temporal acquisition window available for Doppler analysis and motion tracking, increasing susceptibility to frame-rate limitations and motion-related image degradation during deformation imaging [1,2]. Image acquisition may be further compromised by mechanical ventilation, thoracic dressings, pulmonary hyperinflation, and restricted intercostal access in critically ill neonates [3,4]. In fetal cardiovascular imaging, maternal body habitus, fetal motion, placental position, and fetal spine orientation may significantly affect image quality and volumetric reconstruction accuracy [1]. Optimal spatiotemporal image correlation (STIC) acquisition requires favorable fetal positioning and acquisition angles typically ranging between 20° and 40°, whereas fetal movement during volume acquisition may introduce reconstruction artifacts and reduce multiplanar navigation accuracy [1].

In addition to acquisition-related challenges, neonatal echocardiography remains highly dependent on operator expertise during image optimization, Doppler alignment, ventricular segmentation, and functional parameter extraction [8,11]. Interobserver and intraobserver variability continue to affect the reproducibility of ventricular functional assessment and pulmonary hemodynamic measurements, particularly when image quality is suboptimal or geometric assumptions are required. Right ventricular assessment remains especially challenging because of the complex crescentic geometry, trabeculated myocardium, and load-dependent physiology of the neonatal right ventricle [3]. Similarly, Doppler-derived hemodynamic measurements are influenced by insonation angle, sampling position, gain settings, and beat-to-beat variability, introducing additional quantitative inconsistency [2].

Advanced deformation imaging techniques, including tissue Doppler imaging and speckle-tracking echocardiography, provide improved sensitivity for the detection of subtle myocardial dysfunction but remain highly dependent on temporal resolution and tracking stability. Speckle-tracking analysis may be affected by low frame rates, signal dropout, reverberation artifacts, and translational cardiac motion, limiting measurement reproducibility [2]. Three-dimensional and four-dimensional echocardiographic techniques provide improved volumetric visualization compared with conventional two-dimensional imaging but remain vulnerable to motion artifacts, reduced temporal resolution, and increased computational demands [1]. Although STIC-based reconstruction enables offline volumetric analysis and multiplanar navigation, prolonged acquisition intervals may still reduce synchronization accuracy in the presence of fetal movement or rhythm irregularities [1].

Another major limitation involves the lack of universally standardized acquisition and reporting protocols across institutions and ultrasound platforms [6,13]. Variability in ultrasound hardware, transducer frequency, and post-processing software contributes to heterogeneous datasets and may reduce the generalizability of quantitative imaging biomarkers and artificial intelligence models [7,8]. These methodological limitations have accelerated interest in computational image-processing methodologies and AI-assisted analytical pipelines aimed at improving image standardization, reducing observer dependency, and enhancing reproducibility in neonatal cardiovascular ultrasound imaging [12,14].

## 4. Quantitative Echocardiographic Biomarkers and Functional Ultrasound Assessment

The transition from qualitative interpretation toward quantitative cardiovascular ultrasound analysis has become increasingly important in neonatal echocardiography because of the need for objective and reproducible hemodynamic assessment [3]. Commonly used quantitative biomarkers include tricuspid annular plane systolic excursion (TAPSE), fractional area change (FAC), pulmonary artery acceleration time (PAAT), tissue Doppler imaging (TDI), and speckle-tracking echocardiography (STE), which are used to assess ventricular performance, pulmonary hemodynamics, and myocardial deformation [2,3]. TAPSE and FAC are widely used surrogate markers of right ventricular systolic function; however, both remain affected by geometric assumptions, image alignment, loading conditions, and endocardial border delineation [1,3]. Similarly, Doppler-derived parameters such as PAAT and tricuspid regurgitation velocity provide non-invasive estimation of pulmonary hemodynamics but remain dependent on insonation angle, sampling position, and image quality. Tissue Doppler imaging enables quantitative assessment of myocardial velocities with high temporal resolution but remains angle-dependent and susceptible to translational myocardial motion and loading variability [2].

Speckle-tracking echocardiography has emerged as an advanced deformation imaging technique capable of quantifying longitudinal, circumferential, and radial myocardial strain. Because strain imaging evaluates intrinsic myocardial deformation, STE may detect subtle ventricular dysfunction before abnormalities become evident using conventional functional indices [2]. However, STE remains highly dependent on frame rate optimization, tracking stability, and image quality, while significant intervendor variability persists among post-processing software platforms despite ongoing EACVI/ASE standardization initiatives and recent consensus recommendations for deformation imaging [15,16,17]. Quality-assurance analyses using in silico deformation models have further demonstrated persistent variability in segmental strain measurements among commercial speckle-tracking platforms despite ongoing standardization efforts [18]. Similarly, Mirea et al. demonstrated substantial variability in segmental longitudinal strain measurements among commercial software vendors, highlighting the limitations of cross-platform reproducibility in quantitative deformation imaging [19].

Advanced volumetric methodologies, including three-dimensional/four-dimensional echocardiography and spatiotemporal image correlation (STIC), enable multiplanar visualization and quantitative volumetric assessment of fetal cardiac anatomy and function [1]. STIC-based rendering techniques, including tomographic ultrasound imaging, HDlive rendering, and virtual organ computer-aided analysis (VOCAL), further support detailed structural assessment and offline computational analysis [1]. Nevertheless, quantitative echocardiographic imaging continues to face important limitations related to acquisition standardization, reproducibility, temporal synchronization, and cross-platform consistency [2,3]. These methodological limitations highlight the need for increasingly standardized and computationally assisted imaging methodologies in neonatal cardiovascular ultrasound [12,14]. The principal quantitative echocardiographic biomarkers currently used in neonatal and fetal cardiovascular imaging are summarized in Table 2, together with a comparison of their reproducibility, clinical applicability, current clinical evidence, and major methodological limitations.

## 5. Advanced Imaging Methodologies and Volumetric Ultrasound Reconstruction

The transition from conventional two-dimensional echocardiography toward advanced volumetric imaging techniques has substantially expanded the diagnostic capabilities of fetal and neonatal cardiovascular ultrasound assessment by improving spatial visualization and characterization of complex congenital cardiac anatomy [1]. Three-dimensional (3D) and four-dimensional (4D) ultrasound methodologies were introduced to overcome several limitations of conventional two-dimensional imaging, including restricted spatial representation and incomplete assessment of complex intracardiac relationships [1]. In parallel with advances in quantitative echocardiographic imaging and computational analysis, volumetric ultrasound methodologies have increasingly contributed to more comprehensive fetal cardiovascular evaluation [1,3]. Although several of these technologies were originally developed for prenatal cardiac assessment, they are discussed in this review because they have established important methodological principles that have subsequently influenced computational imaging and AI-assisted analysis in neonatal echocardiography.

Among these technologies, spatiotemporal image correlation (STIC) has emerged as a key volumetric imaging modality in fetal cardiology and provides an important technological framework for subsequent advances in computational image analysis that are increasingly relevant to neonatal echocardiography [1,9]. STIC-based imaging permits multiplanar navigation and detailed assessment of cardiac chambers, ventricular septa, atrioventricular valves, outflow tracts, and great vessels while supporting offline post-processing analysis and tele-echocardiography applications [1,10]. Advanced rendering techniques, including tomographic ultrasound imaging (TUI), HDlive rendering, inversion mode, Doppler-integrated reconstruction, and virtual organ computer-aided analysis (VOCAL), further improve structural and volumetric characterization of fetal cardiac anatomy and facilitate enhanced visualization of spatial cardiac relationships [1,10].

Fetal Intelligent Navigation Echocardiography (FINE) has additionally introduced semi-automated generation of standard fetal echocardiographic planes from STIC datasets using intelligent navigation algorithms, thereby reducing operator dependency and improving prenatal cardiac screening workflows [9,10]. Although FINE is currently used almost exclusively in prenatal cardiac imaging, its automated navigation principles have provided the methodological foundation for subsequent advances in AI-assisted image analysis and workflow standardization in neonatal echocardiography. The technological evolution of neonatal and fetal cardiovascular ultrasound imaging is summarized in Figure 1. Advanced volumetric methodologies have proven particularly useful for the evaluation of complex congenital cardiac abnormalities and for multidisciplinary procedural planning using virtual and three-dimensional reconstructed cardiac models [1]. However, volumetric imaging remains highly dependent on acquisition quality, fetal position, temporal synchronization, and motion stability during image capture [1]. Fetal motion, arrhythmias, and unfavorable fetal orientation may introduce reconstruction artifacts that degrade image quality and reduce segmentation accuracy, thereby limiting reproducibility of volumetric analysis [1,2].

In addition, multidimensional ultrasound datasets require substantial computational processing and operator expertise for accurate interpretation. These technical demands have accelerated the integration of artificial intelligence and computational image-analysis frameworks into neonatal cardiovascular imaging workflows, particularly for automated segmentation, image standardization, and quantitative functional assessment [8,12].

## 6. Artificial Intelligence and Computational Ultrasound Analysis

**Figure 2:**
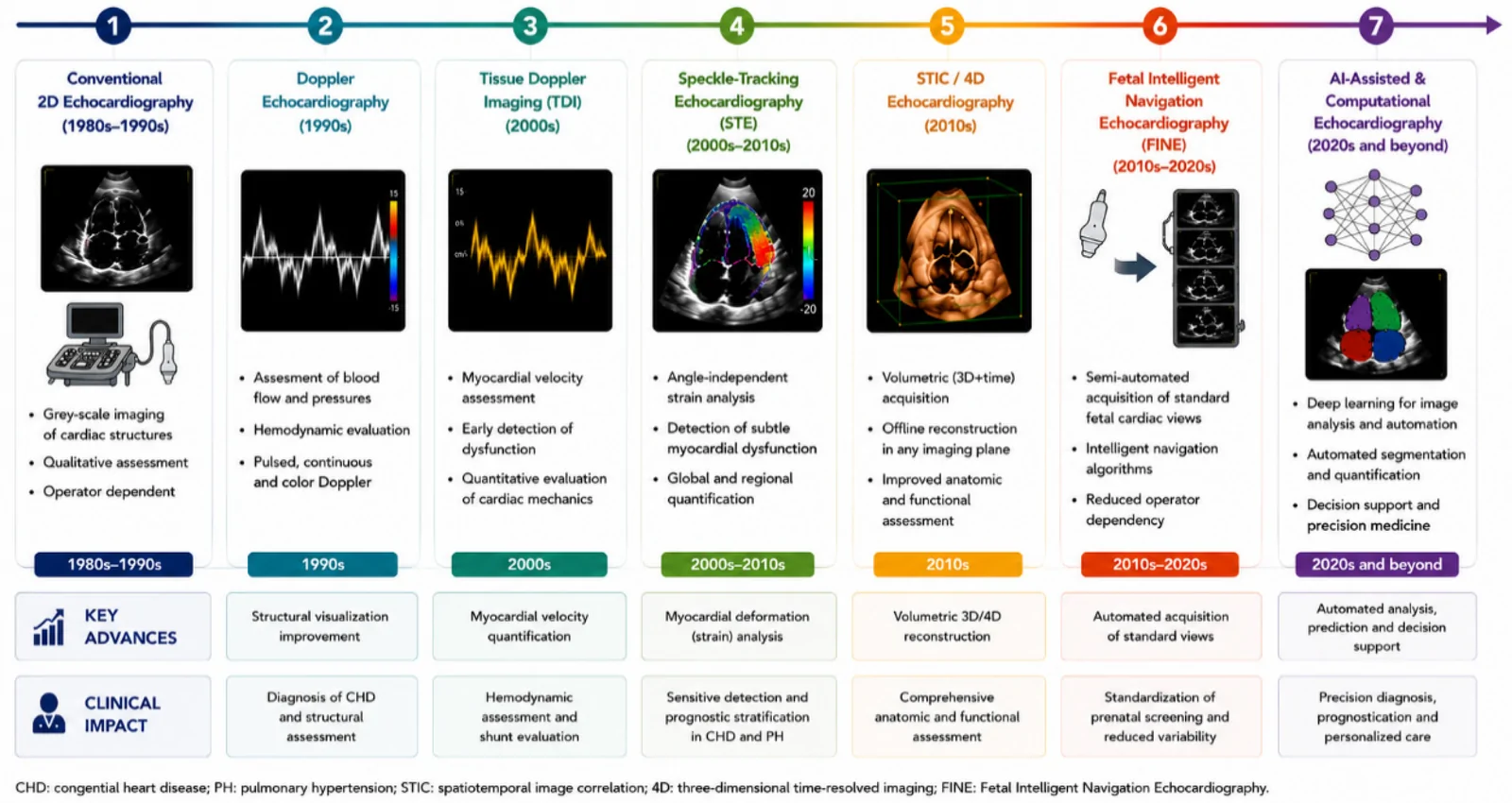
Evolution of neonatal and fetal cardiovascular ultrasound imaging. Timeline illustrating the transition from conventional two-dimensional echocardiography toward advanced quantitative, volumetric, and artificial intelligence-assisted computational imaging methodologies, including Doppler imaging, tissue Doppler imaging, speckle-tracking echocardiography, STIC-based reconstruction, FINE navigation systems, and AI-driven cardiovascular ultrasound analysis.

The increasing complexity of neonatal and fetal cardiovascular ultrasound imaging has accelerated the integration of artificial intelligence (AI), machine learning (ML), and deep learning (DL) methodologies into echocardiographic analysis workflows [8]. AI-assisted pipelines commonly include image preprocessing, automated view classification, chamber segmentation, feature extraction, and quantitative functional assessment [12,14]. As illustrated in Figure 2, contemporary AI-assisted echocardiographic analysis follows a multistage computational workflow that begins with image acquisition and quality control, progresses through automated segmentation and quantitative biomarker extraction, and culminates in AI-assisted interpretation and clinical integration. Representative methodological examples corresponding to each stage of this workflow are discussed below. An overview of the current AI applications in neonatal and fetal echocardiography is presented in Table 3, highlighting representative AI models and architectures, performance metrics, clinical maturity, clinical roles, and current limitations.

The initial stages of the computational pipeline involve image preprocessing, quality control, and automated view classification. Convolutional neural networks (CNNs) represent the dominant deep learning architecture in cardiovascular ultrasound because of their ability to extract hierarchical spatial imaging features from dynamic cardiac datasets [12]. Automated view classification has become particularly important in neonatal and fetal echocardiography because accurate identification of standard imaging planes is essential for reproducible quantitative assessment in critically ill neonates and during prenatal cardiac screening [6,12]. Huang et al. developed a three-dimensional CNN-based model for automated echocardiographic view selection and image-quality assessment using spatiotemporal ultrasound information extracted from moving cardiac sequences, demonstrating high diagnostic performance using classification accuracy and receiver operating characteristic (ROC)-based evaluation metrics [12].

Following image preprocessing, the next stage of the workflow focuses on chamber segmentation and feature extraction. Segmentation algorithms constitute another major component of AI-assisted echocardiography because accurate chamber delineation is required for automated ventricular quantification [12,14]. U-Net-based architectures are commonly used for echocardiographic segmentation, while temporal feature extraction improves automated evaluation of myocardial motion and wall motion abnormalities [12]. The performance of segmentation algorithms is commonly assessed using overlap-based metrics, particularly the Dice similarity coefficient, which has become the standard measure for evaluating automated image segmentation accuracy. However, neonatal and fetal echocardiographic datasets remain relatively limited because of small patient populations, heterogeneous acquisition protocols, and challenges related to image annotation and physiologic variability in early life [7,13].

The development of robust AI models for neonatal echocardiography depends not only on the choice of algorithm but also on the availability of sufficiently large, diverse, and well-curated datasets. Because neonatal and fetal cardiovascular imaging datasets are often relatively limited, multicenter data collection and standardized imaging protocols are essential to improve model generalizability and reduce dataset heterogeneity [7,11,13]. Accurate image annotation by experienced clinicians is equally important for supervised learning, while standardized annotation strategies may improve consistency and reduce interobserver variability during model training [12,14]. In addition, external validation using independent datasets is crucial to evaluate model robustness and generalizability, as reliance on internal validation alone may overestimate diagnostic performance [7,13]. Depending on the intended application, model performance is commonly assessed using sensitivity, specificity, accuracy, the area under the receiver operating characteristic curve (AUC), and, for segmentation tasks, the Dice similarity coefficient. These standardized performance metrics facilitate objective comparison between computational approaches and enable more consistent evaluation of AI systems across different clinical applications [8,12].

Once quantitative imaging features and biomarkers have been extracted, AI models can be applied to disease detection, classification, and risk prediction. AI methodologies have shown promising diagnostic performance in proof-of-concept and early validation studies of prenatal congenital heart disease screening and automated fetal cardiac classification. A recent systematic review and meta-analysis reported pooled sensitivity and specificity values of 0.89 and 0.91, respectively, for AI-assisted prenatal congenital heart disease detection using deep learning-based ultrasound analysis, indicating high overall diagnostic discrimination despite methodological heterogeneity among the included studies [8]. Experimental AI-based computer vision models have also demonstrated encouraging results for automated detection of neonatal cardiovascular abnormalities, including patent ductus arteriosus [20]. Beyond imaging-only applications, AI frameworks have increasingly incorporated multimodal data-integration strategies combining imaging findings with metabolomic and clinical biomarkers for improved prenatal cardiovascular risk prediction [21]. Machine learning approaches have also been applied to automated phonocardiographic signal analysis for structural heart disease screening in pediatric populations [22].

**Figure 3:**
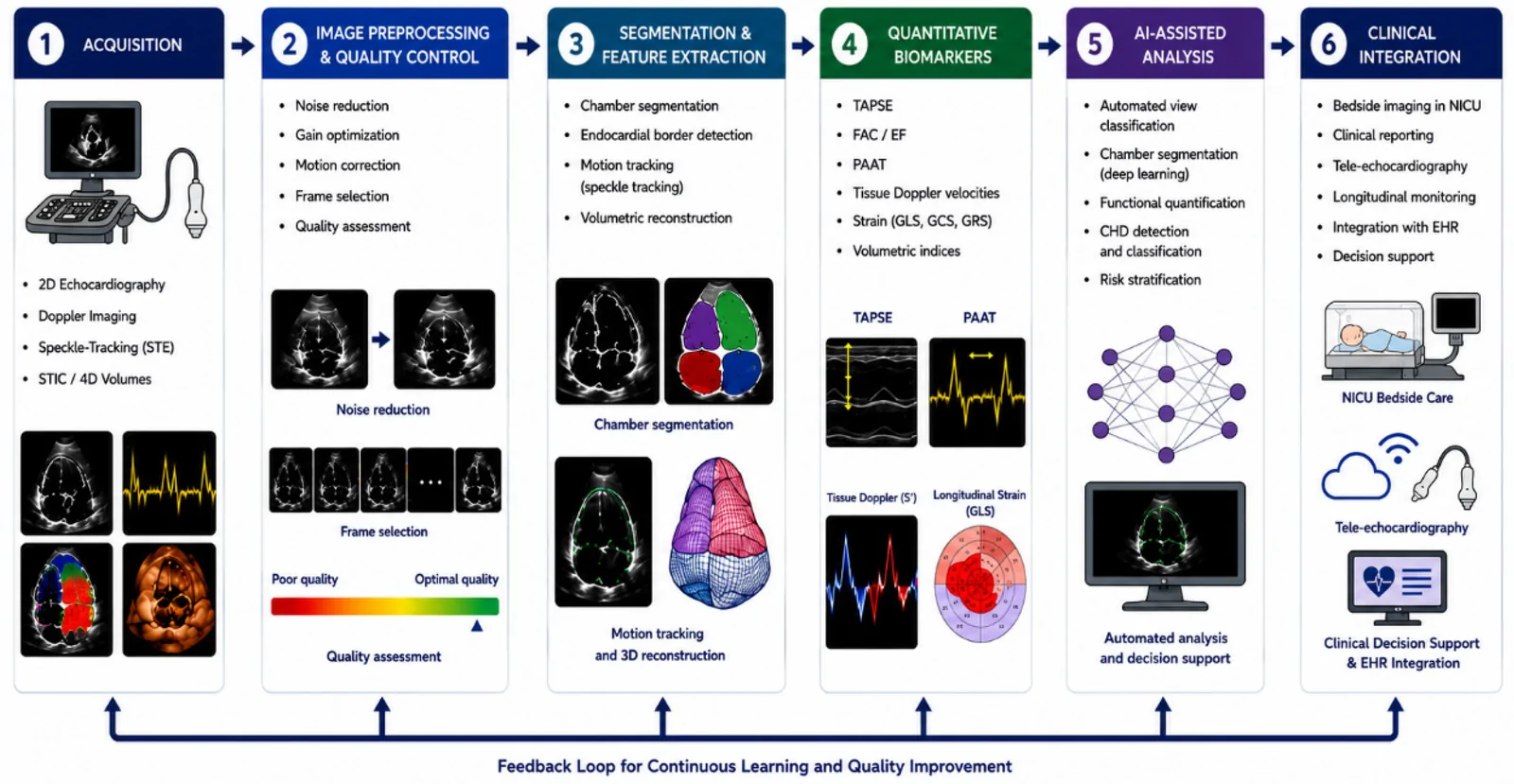
Computational workflow of neonatal and fetal echocardiographic analysis. Schematic representation of the major stages involved in contemporary computational cardiovascular ultrasound imaging, including image acquisition, preprocessing and quality control, chamber segmentation, quantitative biomarker extraction, artificial intelligence-assisted analysis, and clinical integration within neonatal cardiovascular care workflows.

An overview of this computational workflow, from image acquisition and preprocessing to AI-assisted analysis and clinical integration, is illustrated in Figure 3. The final stage of the computational workflow involves the integration of AI-assisted analysis into routine clinical practice and bedside decision-making. Although several AI models have demonstrated promising performance in proof-of-concept and early validation studies, most AI-assisted neonatal echocardiographic systems remain investigational and have not yet undergone sufficient prospective multicenter validation for routine clinical deployment in neonatal intensive care practice. Major limitations continue to restrict broader implementation, including limited dataset size, heterogeneous acquisition protocols, annotation variability, inadequate external validation, explainability concerns, and incomplete integration into real-time clinical workflows [7,13,23]. Explainability has become increasingly important because successful clinical implementation requires interpretable decision-making processes and transparent image-feature analysis [24]. Consequently, further progress in neonatal AI-assisted cardiovascular ultrasound analysis will require standardized datasets, multicenter validation frameworks, explainable AI methodologies, and prospective clinical implementation studies before routine integration into neonatal cardiovascular imaging practice can be achieved [7,8]. Overall, the current body of evidence should be regarded as predominantly comprising proof-of-concept and early validation studies, highlighting the need for robust prospective evaluation before widespread clinical adoption.

## 7. Reproducibility, Standardization, and Validation Challenges

Despite major advances in quantitative ultrasound imaging and artificial intelligence (AI)-assisted analysis, reproducibility and standardization remain major challenges in neonatal and fetal echocardiography [2,8]. Measurement variability may arise at multiple stages of the imaging workflow, including image acquisition, Doppler alignment, segmentation, post-processing, and observer interpretation [3]. Quantitative biomarkers such as strain, TAPSE, FAC, PAAT, and tissue Doppler velocities remain highly dependent on image quality, temporal resolution, and segmentation accuracy, thereby limiting interobserver reproducibility in routine clinical practice [2,3]. As discussed previously, persistent intervendor variability in image acquisition, speckle-tracking software, and post-processing algorithms continues to limit the reproducibility and cross-platform comparability of quantitative deformation imaging despite ongoing EACVI/ASE standardization initiatives [15,16,17,18,19]. Consequently, measurements obtained using different ultrasound systems or software platforms may not be directly interchangeable, potentially affecting longitudinal patient follow-up, multicenter research, and the clinical interpretation of quantitative functional indices.

These methodological limitations become particularly important for AI-assisted echocardiographic analysis because deep learning models are highly sensitive to dataset heterogeneity and image-distribution variability [8]. Most currently available AI models in fetal and neonatal cardiovascular imaging remain limited by relatively small retrospective datasets, restricted external validation, and insufficient multicenter data integration despite recent efforts toward collaborative AI echocardiography initiatives [11]. Variability in ultrasound hardware, acquisition protocols, annotation quality, and patient populations may substantially reduce model generalizability across institutions, potentially resulting in less accurate chamber segmentation, variability in automated ventricular functional measurements, and reduced diagnostic performance when algorithms are applied to external datasets [7,8]. Similarly, limited external validation raises uncertainty regarding algorithm reliability across different clinical settings, ultrasound vendors, and neonatal populations, thereby limiting confidence in routine clinical implementation [7,13]. The absence of universally accepted reference standards for several neonatal hemodynamic biomarkers further complicates both conventional echocardiographic interpretation and supervised AI model development [3].

Successful clinical implementation also requires seamless integration of AI algorithms into real-time bedside acquisition workflows, reporting systems, and neonatal intensive care environments [13]. In addition, regulatory and ethical considerations—including explainability, data privacy, algorithm transparency, and accountability—remain important barriers to widespread clinical adoption of AI-assisted neonatal imaging systems [7,24]. These challenges may reduce clinician confidence in AI-assisted decision support despite encouraging performance reported in proof-of-concept and early validation studies. Consequently, future progress in neonatal computational echocardiography will depend on large multicenter datasets, harmonized acquisition protocols, vendor-independent quantitative standards, robust external validation frameworks, explainable AI methodologies, and prospective implementation studies before routine integration into neonatal cardiovascular imaging practice can be achieved [7,8].

## 8. Future Directions and Emerging Computational Perspectives

Future AI-assisted neonatal echocardiography systems are expected to function increasingly as real-time bedside decision-support tools integrated within neonatal intensive care monitoring ecosystems, reflecting the broader evolution of neonatal echocardiography toward the integration of quantitative ultrasound, artificial intelligence, and multimodal physiologic data analysis [7,11,17,25]. Recent developments suggest a gradual transition from operator-dependent image interpretation toward semi-automated and fully automated echocardiographic workflows incorporating real-time acquisition guidance, automated chamber segmentation, and AI-assisted functional quantification, including automated ventricular functional assessment using deep learning-based echocardiographic analysis [12,17,26].

Emerging machine learning methodologies, including self-supervised and federated learning approaches, have the potential to improve model generalizability while reducing dependence on large manually annotated datasets and facilitating multicenter collaborative training without direct patient-data sharing [7]. However, their successful implementation in neonatal echocardiography remains challenging. Variability in ultrasound equipment, acquisition protocols, image quality, annotation practices, and institutional imaging workflows may result in data drift and heterogeneous data distributions, potentially limiting model performance and generalizability even within federated learning frameworks [7,13]. Consequently, further methodological development and prospective multicenter validation will be required before these approaches can be widely adopted in clinical practice.

Advanced three-dimensional reconstruction and virtual simulation technologies may further support procedural planning and patient-specific cardiovascular assessment in complex congenital heart disease [1,25]. In parallel, portable ultrasound systems, handheld devices, tele-echocardiography platforms, and real-time bedside AI applications may expand access to advanced cardiovascular imaging in neonatal intensive care and resource-limited settings [1,27].

AI-based computer vision models have demonstrated promising performance in the automated detection of neonatal cardiovascular abnormalities, including patent ductus arteriosus [20]. Similar deep learning-based image and video analysis frameworks have also been developed for the prenatal detection of congenital heart defects, highlighting the broader applicability of AI-assisted cardiovascular imaging across the perinatal continuum [28] Machine learning-assisted phonocardiographic analysis has also shown potential for automated screening of congenital heart disease using digital heart sound recordings [22,29]. Recent EACVI/ASE consensus recommendations have additionally emphasized the growing role of automated strain analysis, standardized deformation imaging workflows, and AI-assisted post-processing methodologies in future echocardiographic practice [17].

Despite these promising developments, translation of AI-assisted neonatal echocardiography into routine clinical practice will require continued multidisciplinary collaboration among clinicians, engineers, computer scientists, and industry partners. Future research should prioritize prospective multicenter validation studies, standardized evaluation frameworks, and seamless integration into clinical workflows to ensure that emerging AI technologies are implemented safely, effectively, and equitably in neonatal cardiovascular care [7,24].

## 9. Limitations

This narrative review has several limitations. First, the rapidly evolving nature of artificial intelligence and quantitative cardiovascular imaging research may limit the long-term generalizability of currently available methodologies and reported diagnostic performance. In addition, substantial heterogeneity exists among published studies regarding imaging protocols, ultrasound platforms, segmentation methodologies, quantitative biomarkers, and validation frameworks, limiting direct comparison across studies and reducing cross-platform reproducibility. Many currently available AI-assisted echocardiographic studies remain retrospective, single-center investigations based on relatively small neonatal or fetal datasets, while prospective multicenter validation studies and real-world NICU implementation data remain limited. Furthermore, because this study represents a narrative rather than systematic review, formal risk-of-bias assessment and meta-analytic quantitative synthesis were not performed, introducing the possibility of selection and publication bias. Additional limitations should also be acknowledged. The literature search was restricted to English-language publications, potentially introducing language bias, while the inclusion of Google Scholar may have increased the risk of selection bias because of its broad indexing strategy. Furthermore, unpublished studies and grey literature were not systematically searched, and publication bias cannot be excluded.

## 10. Conclusions

Neonatal echocardiography remains an essential imaging modality for the assessment of congenital and hemodynamic cardiovascular abnormalities. Recent advances in quantitative imaging, volumetric ultrasound reconstruction, and AI-assisted methodologies have considerably expanded the methodological capabilities of neonatal echocardiography and demonstrated the potential to improve diagnostic performance. Nevertheless, important technical and translational challenges remain, including operator dependency, measurement variability, dataset heterogeneity, limited external validation, and incomplete integration into routine clinical workflows, while most AI-assisted echocardiographic systems remain investigational. Continued efforts toward standardized imaging protocols, harmonized quantitative methodologies, multicenter validation studies, explainable artificial intelligence frameworks, and seamless integration into clinical practice will be essential to support the safe and effective adoption of these technologies. As evidence continues to evolve, the integration of quantitative imaging and AI-assisted analysis has the potential to enhance diagnostic consistency, improve clinical decision-making, and contribute to more personalized cardiovascular care for neonates.

## Figures and Tables

**Table 1:** Summary of the literature search strategy and study selection methodology.

Item	Description
Review type	Structured narrative review
Databases searched	PubMed, Scopus, Embase, Google Scholar
Search period	2010–May 2026
Main search concepts	Neonatal echocardiography, fetal echocardiography, cardiovascular ultrasound, AI, machine learning, deep learning, speckle-tracking echocardiography, STIC, congenital heart disease
Language	English
Inclusion criteria	Original articles, methodological studies, clinical investigations and full conference proceedings relevant to neonatal/fetal echocardiography and AI-assisted cardiovascular imaging
Exclusion criteria	Editorials, letters, duplicate publications, case reports without substantial methodological contribution
Initial records	247
Included studies	29
Evidence synthesis	Narrative thematic synthesis

**Table 2:** Comparison of quantitative echocardiographic biomarkers used in neonatal and fetal cardiovascular imaging, including their clinical applicability and current level of clinical evidence.

Biomarker	Clinical Application	Reproducibility/Diagnostic Performance	Clinical Applicability	Current Clinical Evidence	Limitations
TAPSE	Assessment of right ventricular systolic function	Good reproducibility; widely validated for RV systolic assessment	Routine bedside evaluation in NICUs	Established	Angle dependence and load sensitivity
FAC	Global right ventricular functional evaluation	Moderate reproducibility; affected by endocardial border delineation	Commonly used for global RV assessment	Established	Image-quality dependence
PAAT	Estimation of pulmonary hemodynamics	Useful surrogate marker; influenced by Doppler acquisition	Assessment of pulmonary vascular resistance	Established	Doppler angle dependency and sampling variability
TDI	Quantitative myocardial velocity assessment	High temporal resolution but angle dependent	Evaluation of myocardial velocities and diastolic function	Widely adopted	Translational motion artifacts
STE	Myocardial deformation and strain analysis	High sensitivity for subclinical dysfunction; limited cross-vendor reproducibility	Advanced myocardial functional assessment	Emerging	Vendor variability, frame-rate dependence, and tracking instability
STIC	Volumetric fetal cardiac assessment	Enables comprehensive volumetric evaluation; reproducibility depends on acquisition quality	Detailed fetal cardiac anatomy and offline analysis	Established in fetal imaging; limited neonatal application	Motion artifacts and temporal synchronization limitations

Abbreviations: FAC, fractional area change; PAAT, pulmonary artery acceleration time; RV, right ventricle/right ventricular; STE, speckle-tracking echocardiography; STIC, spatiotemporal image correlation; TAPSE, tricuspid annular plane systolic excursion; TDI, tissue Doppler imaging.

**Table 3:** Representative artificial intelligence models, performance metrics, clinical maturity, and applications in neonatal and fetal echocardiography.

AI Application	Representative AI Models/Architectures	Representative Performance Metrics	Clinical Maturity	Clinical Role	Current Limitations
Automated view classification	CNN-based models (including 3D CNNs)	Classification accuracy; ROC-AUC	Early clinical validation	Identification of standard echocardiographic planes	Limited external validation and dataset heterogeneity
Chamber segmentation	U-Net-based architectures	Dice similarity coefficient	Early clinical validation	Automated ventricular quantification and border detection	Annotation variability and image-quality dependence
Congenital heart disease detection	Deep CNN classifiers	Sensitivity 89%; Specificity 91% (meta-analysis)	Advanced prenatal validation; limited neonatal implementation	Prenatal CHD screening and diagnostic support	Limited multicenter validation and imbalanced datasets
Image quality assessment	CNN-based quality assessment models	Classification accuracy	Early clinical validation	Standardization of image acquisition workflows	Vendor-specific variability and generalizability limitations
Strain and deformation analysis	Motion-tracking and deep learning regression models	Correlation coefficient; Mean absolute error	Investigational	Automated myocardial functional assessment	Intervendor reproducibility limitations and frame-rate dependency
Tele-echocardiography and remote analysis	AI-assisted decision-support systems	Clinical feasibility; Diagnostic agreement	Emerging	Remote cardiovascular assessment and NICU support	Workflow integration, bandwidth, and data privacy concerns

Clinical maturity reflects the current stage of clinical translation based on the available literature and does not represent a formal technology readiness classification. Abbreviations: AI, artificial intelligence; AUC, area under the receiver operating characteristic curve; CHD, congenital heart disease; CNN, convolutional neural network; NICU, neonatal intensive care unit; ROC, receiver operating characteristic; U-Net, U-shaped convolutional neural network architecture.

## Data Availability

No new data were created or analyzed in this study. Data sharing is not applicable to this article.

## Abbreviations

AI	Artificial intelligence
AUC	Area under the receiver operating characteristic curve
CHD	Congenital heart disease
CNN	Convolutional neural network
DL	Deep learning
FAC	Fractional area change
FINE	Fetal Intelligent Navigation Echocardiography
ML	Machine learning
NICU	Neonatal intensive care unit
PAAT	Pulmonary artery acceleration time
RV	Right ventricle/right ventricular
STE	Speckle-tracking echocardiography
STIC	Spatiotemporal image correlation
TAPSE	Tricuspid annular plane systolic excursion
TDI	Tissue Doppler imaging
TNE	Targeted neonatal echocardiography
TUI	Tomographic ultrasound imaging
U-Net	U-shaped convolutional neural network architecture
VOCAL	Virtual Organ Computer-aided Analysis
